# Synaptosomal-Associated Protein 25 Gene Polymorphisms Affect Treatment Efficiency of Methylphenidate in Children With Attention-Deficit Hyperactivity Disorder: An fNIRS Study

**DOI:** 10.3389/fnbeh.2021.793643

**Published:** 2022-01-05

**Authors:** Jie Li, Wen-Jie Yan, Yan Wu, Xin-Xin Tian, Yi-Wen Zhang

**Affiliations:** ^1^Department of Developmental and Behavioral Pediatrics, Shanghai Children's Medical Center, School of Medicine, Shanghai Jiao Tong University, Shanghai, China; ^2^Department of Pediatrics, Sichuan Academy of Medical Sciences and Sichuan Provincial People's Hospital, Chengdu, China

**Keywords:** functional near-infrared spectroscopy, ADHD, methylphenidate, SNAP-25, polymorphisms

## Abstract

Methylphenidate (MPH) is the first-line drug for the treatment of children with attention-deficit hyperactivity disorder (ADHD); however, individual curative effects of MPH vary. Many studies have demonstrated that synaptosomal-associated protein 25 (*SNAP-25*) gene *Mnl*I polymorphisms may be related to the efficacy of MPH. However, the association between *SNAP-25Mnl*I polymorphisms and changes in brain hemodynamic responses after MPH treatment is still unclear. This study used functional near-infrared spectroscopy (fNIRS) to preliminarily investigate the interaction of MPH treatment-related prefrontal inhibitory functional changes with the genotype status of the *SNAP-25* gene in children with ADHD. In total, 38 children with ADHD aged 6.76–12.08 years were enrolled in this study and divided into the following two groups based on *SNAP-25* gene *Mnl*I polymorphisms: T/T genotype group (wild-type group, 27 children) and G allele carrier group (mutation group, 11 children). The averaged oxygenated hemoglobin concentration changes [Δavg oxy-Hb] and deoxyhemoglobin concentration changes [Δavg deoxy-Hb] in the frontal cortex before MPH treatment and after 1.5 h (post-MPH_1.5h_) and 4 weeks (post-MPH_4w_) of MPH treatments were monitored using fNIRS during the go/no-go task. SNAP-IV scores were evaluated both pre-MPH and post-MPH_4w_ treatments. In the T/T genotype group, [Δavg oxy-Hb] in the dorsolateral prefrontal cortex was significantly higher after 4 weeks of MPH (post-MPH_4W_) treatment than pre-treatment; however, in the G allele group, no significant differences in [Δavg oxy-Hb] were observed between pre- and post-treatments. In the go/no-go task, the accuracy was significantly increased post-MPH_4w_ treatment in the T/T genotype group, while no significant differences were observed in response time and accuracy of the “go” sand no-go task in the G allele group for pre-MPH, post-MPH_1.5h_, and post-MPH_4w_ treatments. The T/T genotype group exhibited a significant decrease in SNAP-IV scores after MPH treatment, while the G allele group showed no significant difference. In conclusion, fNIRS data combined with *SNAP-25 Mnl*I polymorphism analysis may be a useful biomarker for evaluating the effects of MPH in children with ADHD.

## Introduction

Attention-deficit hyperactivity disorder (ADHD) is a common neurodevelopmental disorder characterized by persistent inattention, hyperactivity, and impulsivity (Posner et al., 2020). ADHD is associated with functional deficits in children, including poor interpersonal relationships (especially parent-child and sibling relationships), poor academic performance, low self-evaluation, and negative emotions. Additionally, children with ADHD are more likely to develop anxiety, depression, and other mental disorders. Notably, ADHD symptoms can persist into adulthood, which can negatively impact the patient's physical and mental health, family life, and social skills in adulthood (Banaschewski et al., 2017). The global prevalence of ADHD in children and adolescents is ~6.29% (Posner et al., 2020). Consistently, a recent meta-analysis of the prevalence of ADHD among school-age children and adolescents in China reported a total prevalence of 6.3% (Liu et al., 2018). ADHD symptoms are associated with alterations in the prefrontal cortex (PFC) and subcortical areas and are thought to be underscored by impaired neurotransmission and insufficient catecholamine production. Although the etiology of ADHD remains poorly understood, empirical evidence suggests that the symptoms improve after drug treatment. Currently approved first-line drug therapies for the treatment of ADHD include psychostimulants, such as amphetamine and methylphenidate (MPH). Tomoxetine is the first non-stimulant drug approved for treating ADHD. MPH is a catecholamine agonist that blocks dopamine (DA) and noradrenaline (NE) transporters, which regulate the normal reuptake of neurotransmitters. Stimulant drugs increase the levels of extracellular DA and NE in synapses in the PFC and striatum, thereby restoring executive function (Caye et al., 2019). However, considerable inter-individual differences exist in clinical results, optimal drug dosages, and duration of effects, which may reflect genetic effects.

Indeed, studies on pharmacogenetic predictors of the efficacy of MPH for treating ADHD have demonstrated that gene polymorphisms, including *SLC6A2, SLC6A3, COMT, DRD4, ADRA2A*, and *SNAP-25*, may be related to the efficacy of MPH (Gomez-Sanchez et al., 2017; Myer et al., 2018). Notably, synaptosomal-associated protein 25 (SNAP-25) is a presynaptic plasma membrane protein that docks in vesicles. SNAP-25 plays a key role in vesicle fusion mechanisms, thereby regulating the release of neurotransmitters from the presynaptic membrane into the synaptic cleft. In addition, SNAP-25 is involved in axon growth and synaptic plasticity (Pozzi et al., 2019). For example, the decreased expression of SNAP-25 mRNA and protein in model mice lacking SNAP-25 (50% lower than that in wild-type mice) leads to symptoms of hyperactivity (Corradini et al., 2009), and the transgenic repair of SNAP-25 function restores normal dopaminergic transmission (Steffensen et al., 1999). These findings suggest that the *SNAP-25* gene may underpin hyperactive behavior.

The 1065 T > G single nucleotide polymorphism (SNP) of the *SNAP-25* gene occurs due to a change of 1065 T to G, which leads to an increase in the restriction site of *Mnl*I, resulting in *SNAP-25* gene *Mnl*I polymorphism (rs3746544). Three genotypes (T/T, T/G, and G/G) have been identified. A study of 165 preschool children with ADHD showed that children with the G allele in *SNAP-25* gene *Mnl*I polymorphism were 2–3 times more likely to be irritable and had more sleep problems during MPH treatment than T carriers (McGough et al., 2006). In another study of children with ADHD, among children with 1065 T > G genotypes, 33.3% of children with the G/G genotype responded well to MPH, whereas 74.7% of children with T/T and 72.5% of those with T/G genotypes responded well to MPH treatment (Song et al., 2014). These studies indicate that *SNAP-25* gene *Mnl*I polymorphisms may be related to the efficacy and adverse reactions of MPH in ADHD children.

Functional near-infrared spectroscopy (fNIRS) is an optical imaging technique that uses near-infrared light to penetrate the skull. This technique takes advantage of the differences in absorption of infrared light by hemoglobin in the blood, and changes in hemoglobin in different cortical areas are used as a proxy of brain activation (Pinti et al., 2020). The infrared rays used in the device are present in sunlight and do not cause physical damage. Compared with functional magnetic resonance imaging (fMRI), fNIRS has advantages in the research field of childhood ADHD: it is quiet and does not require patients to remain still, and the environment is noiseless. Notably, fNIRS has been widely implemented in various research fields. Several studies have employed fNIRS to monitor the effects of drug therapy on cortical hemodynamics and suggested that fNIRS data can be used as a biomarker for drug therapy outcomes (Grazioli et al., 2019; Chen et al., 2020).

To date, it remains unclear whether the changes in brain function differ between genotypes of *SNAP-25* gene *Mnl*I polymorphism in children with ADHD after MPH treatment. Öner et al. (2011) used near-infrared brain function monitoring to examine brain function before and after MPH treatment in children with ADHD during the go/no-go task. They found that the *SNAP-25 Mnl*I genotype was significantly associated with changes in right prefrontal oxyhemoglobin (HbO_2_) and left prefrontal deoxyhemoglobin (HHb) after MPH treatment, and the mean left prefrontal HHb increased during MPH treatment in participants with *Mnl*I T/G and G/G genotypes and decreased in participants with the T/T genotype; the reverse pattern was observed for right prefrontal HbO_2_, which was increased in the T/T group and decreased in the T/G and G/G groups. However, only 16 children with ADHD were enrolled in the study, and 6 of them had other comorbidities, including anxiety disorders, elimination disorders, depression, and other behavioral disorders. These factors might have impacted near-infrared detection of cerebral cortical blood flow. Indeed, in that study, short-term (24 h) cerebral function was monitored after MPH treatment, but the changes in near-infrared cortical blood flow were not monitored after long-term MPH treatment. As such, studies with a larger sample size and fewer confounders are needed to analyze long-term fNIRS cerebral blood flow changes. Accordingly, this study aimed to explore possible pharmacogenetic predictors of MPH treatment outcomes in children with ADHD using near-infrared spectral imaging technology combined with *SNAP-25 Mnl*I polymorphism detection to provide a reference for the clinical prediction of drug efficacy. We hypothesized that in ADHD children, the efficacy of MPH and the changes in near-infrared brain function before and after MPH treatments might be related to *SNAP-25* gene *Mnl*I polymorphism.

## Materials and Methods

### Participants

A total of 45 right-handed children newly diagnosed with ADHD (age range, 6.76–12.08 years; mean age, 8.77 ± 1.16 years) were recruited at the Department of Developmental and Behavioral Pediatrics of Shanghai Children's Medical Center. The diagnosis of ADHD was confirmed by two experienced developmental-behavioral pediatricians according to the Diagnostic and Statistical Manual of Mental Disorders, 5th edition (Posner et al., 2020). The inclusion criteria for children with ADHD were (1) 6–12 years of age, (2) right-handedness confirmed using the Edinburgh Handedness Inventory (Oldfield, 1971), and (3) IQ > 70 based on the Chinese version of the Wechsler Intelligence Scale for Children-Second Edition. The exclusion criteria were the presence of (1) comorbid disorders other than oppositional defiant and conduct disorders; (2) a history of neurological diseases such as epilepsy, cerebral palsy, or brain injury; or (3) serious medical conditions or a history of substance abuse or dependence. Of the 45 children with ADHD, 4 children withdrew from the study due to intolerable adverse reactions (nausea, vomiting, and dizziness) to MPH, and 3 children missed follow-up for personal reasons; finally, a total of 38 children (age range, 6.76–12.08 years; mean age, 8.72 ± 1.16 years) completed the follow-up study. Due to the small number of participants with the SNAP-25 G/G genotype, based on the presence of the rare G allele, the sample was divided into two groups: T/T genotype (27 children) and G allele carriers (T/G + G/G, 11 children) (Kim et al., 2017). The number of participants required for adequate statistical power was based on previous studies that investigated drug effects in ADHD patients using fNIRS (Monden et al., 2012a; Matsuura et al., 2014; Sanefuji et al., 2014).

The present study was approved by the Ethics Committee of Shanghai Children's Medical Center, School of Medicine, Shanghai Jiao Tong University (SCMCIRB-K2021045-1). Written informed consent was obtained from all children and one of their parents prior to the study.

### Research Methods

Blood samples were collected to detect *SNAP-25* gene *Mnl*I polymorphisms in 45 children with ADHD who met the inclusion criteria. Based on the test results, children were divided into two groups (T/T genotype group and G allele carrier group). All participants were MPH-naïve and commenced MPH treatment (18 mg/day) in the morning (Posner et al., 2020). Changes in prefrontal oxygenated hemoglobin and deoxyhemoglobin levels during the go/no-go task were monitored using fNIRS in the drug-naïve condition (pre-treatment), after 1.5 h and 4 weeks (post-MPH_1.5h_ and post-MPH_4w_ treatment) of MPH treatment with the osmotic release oral system (Concerta, Xi'an-Janssen Pharma, XiAn, China). Assessment of brain function in the fourth week (post-MPH_4w_ treatment) was also performed 1.5 h after oral MPH. The primary outcome measures for assessing ADHD symptoms were the four SNAP (Swanson, Nolan, and Pelham)-IV subscales (total score, 26 items; incentive score, 9 items; hyperactive/impulsive score, 9 items; and oppositional score, 8 items). Oppositional scores were included because an oppositional defiant disorder is often present in children with ADHD (Correia Filho et al., 2005). The snap-IV questionnaire was completed by the same parent before and after 4 weeks of MPH treatment.

### Molecular Analysis

Venous blood was collected in tubes containing ethylenediaminetetraacetic acid. DNA was extracted from whole blood using a TIANamp Genomic DNA Kit (TIANGEN, cat. no.: DP304). Polymerase chain reaction (PCR)-restriction fragment length polymorphism assays were used to determine *SNAP-25* gene (GenBank Accession Number D21267) *Mnl*I (rs3746544) polymorphisms. The oligonucleotide primers used to determine the *Mnl*I polymorphisms within the *SNAP-25* gene have been described previously (Yang et al., 2020). The primers used to amplify the *SNAP-25* gene (Yang et al., 2020) were as follows: forward, 5-TTCTCCTCCAAATGCTGTCG-3 and reverse, 5-CCACCGAGGAGAGAAAATG-3. PCR was performed in a 30-μL volume with 100 ng DNA, 20 pmol of each primer, 15 μL of 2 × Hieff PCR Master Mix (With Dye) (Yeason, cat. no.: 10102ES03), and ddH_2_O. Amplification was performed using an automated thermal cycler (Techne Flexigene, Cambridge, UK). PCR conditions were as follows: 5 min for initial denaturation at 95°C, 35 cycles at 95°C for 45 s for denaturation, 1 min at 58°C for annealing, and 1 min at 72°C for extension, followed by 7 min at 72°C for final extension.

### fNIRS Procedure

**Figure 1:**
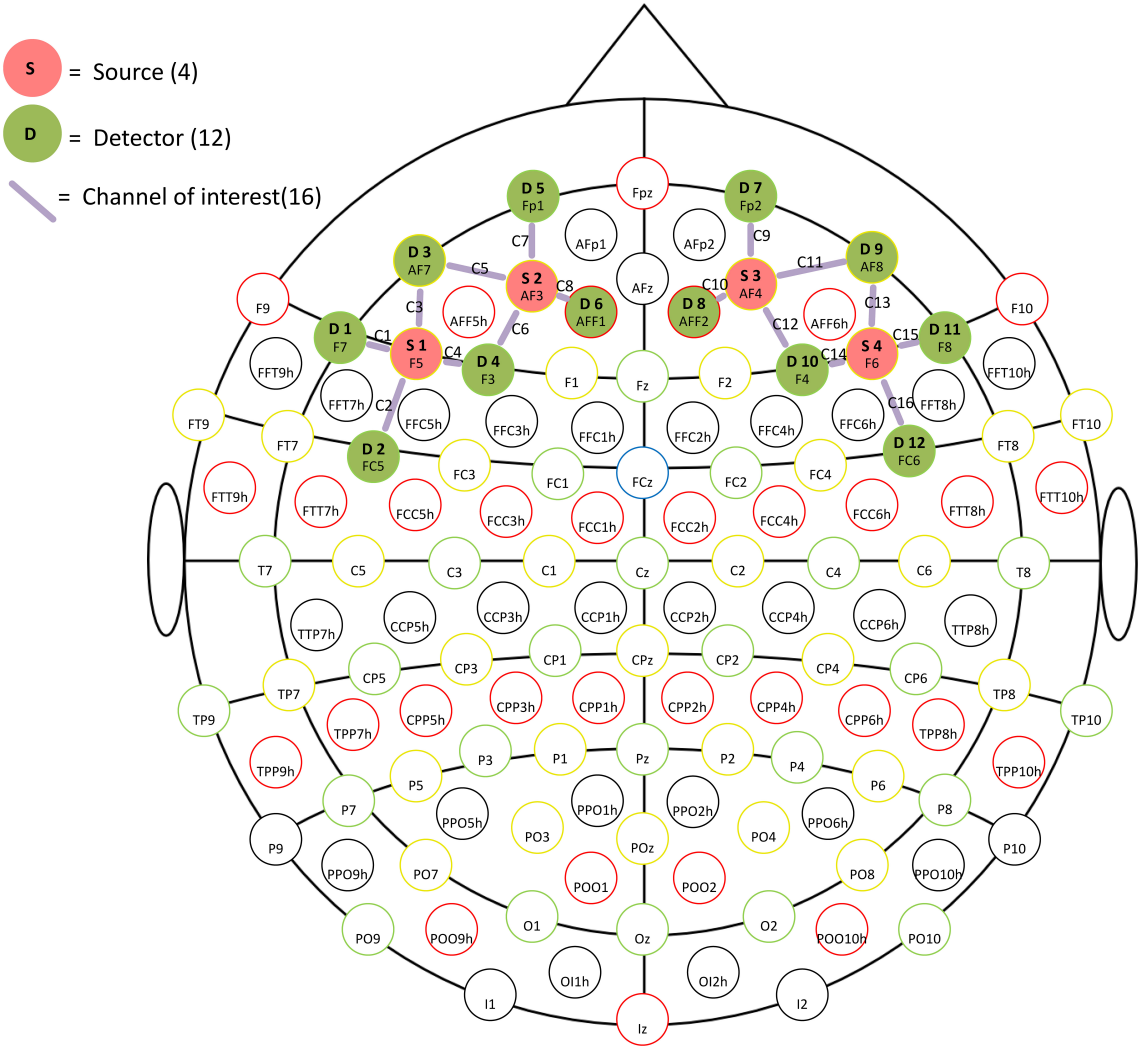
The head probe placed on the forehead. The letters D and S represent the detectors and light source, respectively. The mauve lines with the letter C represent channels.

Data of prefrontal Δavg oxy-Hb and Δavg deoxy-Hb were acquired using a continuous wave near-infrared spectroscopy device, the NIRSport near-infrared imager (NIRSport, NIRx Medical Technology LLC, Glen Head, NY, United States). This device is capable of transmitting near-infrared light at two wavelengths of 785 and 830 nm. The fNIRS device consists of 4 sources and 12 detectors, which have 16 channels that cover the bilateral prefrontal areas (Figure 1), with a constant source-detector distance of 3 cm and a sampling rate of 15.625 Hz. A standard montage was created and adapted for each helmet based on the international 10-20 transcranial positioning system. The sources and detectors were mounted in a pre-selected montage using an EasyCap (https://pressrelease.brainproducts.com/easycap-cap-overview/, EasyCap, Germany).

Figure 1 depicts the approximate placement of the head probe on the forehead. The base of the probe was positioned to align with the eyebrows, while the midline of the probe was aligned with the middle of the forehead. Detectors 1–4 and 9–12 scanned the left and right dorsolateral prefrontal cortex (DLPFC), respectively (Katagiri et al., 2010).

The data were analyzed by nirsLAB (version v2017.06, NIRx Medical Technologies, Glen Head, NY, USA) and MatLab version 2019a (The Mathworks, USA). Individual timeline data for the optical density signals in each channel were preprocessed to eliminate discontinuities and remove spikes due to head movements. Then, the artifact-free data were filtered with a high-pass filter using cut-off frequencies of 0.01 Hz to remove baseline drift and a 0.8 Hz low-pass filter, as well as heartbeat pulsations; subsequently, the modified Beer–Lambert Law (Hoshi, 2007) was used to convert light intensities into concentration changes in oxygenated hemoglobin ([Δoxy-Hb]) and deoxyhemoglobin ([Δdeoxy-Hb]). The[Δavg oxy-Hb] and [Δavg deoxy-Hb] during the go/no-go blocks with go blocks as baselines were calculated in each channel after block averaging of multiple trials.

### Go/No-Go Task

Each session consisted of six block sets, and each set contained alternating go (baseline) and go/no-go (target) blocks. Each block, consisting of 12 trails with a picture presented for 800 ms followed by an inter-stimulus interval for 200 ms, lasted 24 s and was preceded by instructions displayed for 3 s, resulting in an overall block-set time of 54 s and a total session time of 5 min 24 s. In the go block, we presented participants with a random sequence of two pictures and instructed them to press a button for both pictures. In the go/no-go block, we presented participants with a no-go picture for 50% of the time, thus requiring participants to respond to half the trials (go trials) and inhibit their response to another half (no-go trials). A go/no-go ratio of 50% was selected because this was the most commonly used ratio in previous neuroimaging studies (Monden et al., 2012a,b; Nagashima et al., 2014). Each participant performed a practice block before any measurements to ensure that they understood the instructions.

### Behavioral Data Analysis

We calculated the average response times (RTs) for go trials and accuracy rates for go and no-go trials in each go/no-go block in children with ADHD. The accuracy and RTs were averaged across go/no-go blocks, and the resulting values were subjected to statistical analyses as described in the subsequent section. The accuracy for go and no-go trials was computed by dividing the number of correct responses or inhibitions (i.e., participants pressed the button in go trials and did not press the button in no-go trials) by the total number of go trials for the go/no-go block.

### Statistical Analysis

Data were analyzed using IBM SPSS Statistics version 22. The Kolmogorov–Smirnov test was used to assess normality of data. The Chi-squared test was used to compare categorical data. For normally distributed data, we performed two-tailed paired *t*-tests to compare post-MPH_4w_ treatment and pre-MPH treatment values. The data of pre-MPH treatment, post-MPH_1.5h_ treatment, and post-MPH_4w_ treatment were analyzed by one-way ANOVA. Continuous variables and independent samples were compared using an independent samples *t*-test. For non-normally distributed data, the Wilcoxon signed rank test was used to assess independent samples, and the data of pre-MPH treatment, post-MPH_1.5h_ treatment, and post-MPH_4w_ treatment were analyzed by the Kruskal–Wallis one-way ANOVA. In addition, the Mann–Whitney U-test was used to compare data between groups. Sex, age, therapy time, genotype, [Δoxy-Hb], and [Δdeoxy-Hb] in each channel in pre-MPH, post-MPH_1.5h_, and post-MPH_4w_ treatments were analyzed using a generalized linear mixed model (GLMM) (von Lühmann et al., 2020). Statistical significance was set at *p* < 0.05.

## Results

### Demographic Characteristics

Demographic characteristics of all enrolled patients are presented in Table 1. No significant differences were observed in mean age, sex, FIQ, or SNAP-IV scores between the T/T genotype and G allele carrier groups (*p* > 0.05 for all variables, Table 2).

**Table 1:** Demographic and clinical characteristics of 45 recruited children and 38 included children with attention-deficit hyperactivity disorder.

**Variables**	**Recruited children (*n* = 45)**	**Included children (*n* = 38)**	***X*** ^**2**^ ***/* *t***	***p*** **-values**
Male [*n* (%)]	36 (80)	29 (76.4)	0.16	0.68
Age (Mean years ± SD)	8.77 ± 1.16	8.72 ± 1.16	0.19	0.85
FIQ (Mean ± SD)	93.3 ± 10.8	93.1 ± 11.0	0.14	0.89
**SNAP-IV (Mean** **±** **SD)**				
SNAP-IV IA	1.93 ± 0.42	1.95 ± 0.39	−0.17	0.86
SNAP-IV IH	1.41 ± 0.60	1.39 ± 0.64	0.09	0.93
SNAP-V ODD	1.38 ± 0.64	1.36 ± 0.68	0.1	0.92
**Maternal educational level**				
Higher education	21 (46.6%)	17 (44.7%)	0.03	0.86
Secondary education	24 (53.3%)	21 (55.3)		
**Annual family income (10,000 RMB/year)**				
30–100	19 (42.2%)	15 (39.5%)	0.07	0.97
15–30	18 (40%)	16 (42.1%)		
5–15	8 (17.7%)	7 (18.4%)		
Only child in the family [*n* (%)]	28 (62.2)	24 (63.2)	0.17	0.68
T/T genotype number	33 (73.3%)	27 (71.1%)	0.05	0.82
G allele carrier number	12 (26.7)	11 (28.9%)		

*FIQ, full-scale IQ; WISC-II, Wechsler Intelligence Scale for Children's Fourth Edition; ARF, ADHD RS IV-J full scores; SNAP-IV IA, inattention subscale scores; SNAP-IV IH, hyperactivity subscale scores; SNAP-IV ODD, oppositional defiance*.

**Table 2:** Comparison of basic information in the two genotype groups.

	**T/T genotype group**		**G allele carrier group**		***X*** ^**2**^ ***/* *t***	***P*** **-values**
	**Mean**	**SD**	**Mean**	**SD**		
Sex (male/female)	21/6		8/3		0.11	0.52^a^
Age (years)	8.8	1.2	8.5	1.08	0.57	0.53^b^
FIQ (WISC-II)	94.3	10.7	90.2	11.7	0.59	0.30^b^
SNAP-IV IA	1.95	0.42	1.93	0.32	0.17	0.86^b^
SNAP-IV IH	1.50	0.60	1.14	0.68	1.65	0.11^b^
SNAP-V ODD	1.50	0.65	1.02	0.66	2.05	0.05^b^

*FIQ, full-scale IQ; WISC-II, Wechsler Intelligence Scale for Children's Fourth Edition; ARF, ADHD RS IV-J full scores; SNAP-IV IA, inattention subscale scores; SNAP-IV IH, hyperactivity subscale scores; SNAP-IV ODD, oppositional defiance*.

a *Chi-square test*.

b *Independent samples t-test*.

### Comparison of fNIRS Measurements Between Pre- and Post-MPH Treatments

The GLMM showed that [Δavg oxy-Hb] in channels 4, 10, and 12 were significantly higher post-MPH_4w_ treatment than pre-MPH treatment in the T/T genotype group (*t* = −2.36, −2.17, and −2.59, respectively; *p* = 0.02, 0.03, and 0.01, respectively) (Table 3). In the G allele carrier group, [Δavg oxy-Hb] in channels 4, 10, and 12 were not significantly different between the post-MPH_4w_ and pre-MPH conditions (*t* = −1.26, −1.39, and −1.08, respectively; *p* = 0.21, 0.17, and 0.28, respectively) (Table 3). There were no significant differences in sex, age, genotype, treatment time, and [Δavg deoxy-Hb] of each channel among different conditions (*p* > 0.05). Channel 10 was located in the border region between the right DLPFC and frontal eye fields based on the macroanatomical brain atlases (Shattuck et al., 2008). Channels 4 and 12 were located in the left and right DLPFC, respectively. After 4 weeks of MPH treatment, the T/T group exhibited higher prefrontal activation during go/no-go tasks, whereas the T/G group did not exhibit activation in both prefrontal areas, suggesting that *SNAP-25* gene *Mnl*I polymorphisms may be associated with changes in brain function after MPH treatment.

**Table 3:** Comparison of [Δavg oxy-Hb] between pre- and post-MPH treatment conditions in the two groups.

**Genotype**		**T/T genotype group**			**G allele carrier group**		
		**[Δavg oxy-Hb] (mM-mm)**			**[Δavg oxy-Hb] (mM-mm)**		
**Channel**		**Channel 10**	**Channel 12**	**Channel 4**	**Channel 10**	**Channel 12**	**Channel 4**
Pre-MPH therapy		−11.6 × 10^−5^ (7.64 × 10^−5^)	4.64 × 10^−5^ (9.58 × 10^−5^)	−11.3 × 10^−5^ (10.4 × 10^−5^)	−10.1 × 10^−5^ (12.0 × 10^−5^)	8.73 × 10^−5^ (15.0 × 10^−5^)	−11.9 × 10^−5^ (16.3 × 10^−5^)
Post-MPH_1.5h_ therapy		4.18 × 10^−5^ (7.64 × 10^−5^)	25.4 × 10^−5^ (12.9 × 10^−5^)	8.16 × 10^−5^ (10.4 × 10^−5^)	4.84 × 10^−5^ (12.0 × 10^−5^)	9.43 × 10^−5^ (20.2 × 10^−5^)	14.8 × 10^−5^ (16.3 × 10^−5^)
Post-MPH_4w_ therapy		11.8 × 10^−5^ (7.64 × 10^−5^)	30.4 × 10^−5^ (9.58 × 10^−5^)	29.5 × 10^−5^ (13.9 × 10^−5^)	13.5 × 10^−5^ (12.0 × 10^−5^)	31.6 × 10^−5^ (15.0 × 10^−5^)	22.3 × 10^−5^ (21.7 × 10^−5^)
Pre-MPH vs. post-MPH_1.5h_	*t*	−1.11	−1.87	−1.33	−0.88	−0.03	0.13
	*P*	0.27	0.06	0.19	0.38	0.98	0.90
Pre-MPH vs. post-MPH_4w_	*t*	−2.17	−2.59	−2.36	−1.39	−1.08	−1.26
	*P*	0.03^*^	0.01^*^	0.02^*^	0.17	0.28	0.21
Post-MPH_1.5h_ vs. post-MPH_4w_	*t*	−1.05	−0.31	−1.23	−0.99	−1.08	−1.37
	*P*	0.29	0.76	0.22	0.61	0.28	0.17

*MPH, methylphenidate*.

* *p < 0.05*.

### Clinical Outcomes and Task Performance

No significant differences were observed in SNAP-IV scores, go accuracy, no-go accuracy, and go RTs between the T/T genotype and G allele groups in the pre-MPH or post-MPH treatment conditions. A separate analysis of each group revealed that in the T/T group, SNAP-IV scores, including inattention subscale scores, hyperactivity subscale scores, and oppositional defiance subscale scores, were significantly lower in the post-MPH_4w_ treatment condition than in the pre-treatment condition (*p* = 0.001, 0.003, and 0.001, respectively) (Table 4). In the G allele carrier group, no significant differences were observed in SNAP-IV scores, go accuracy, no-go accuracy, or go RTs between pre-MPH treatment and post-MPH_1.5h_ or post-MPH_4w_ treatment conditions (*p* > 0.05 for all variables). SNAP-IV (Hall et al., 2020), rated by parents, has been widely used to monitor the efficacy of MPH treatment. These results suggest that the T/T genotype may be associated with better clinical effects of MPH treatment.

**Table 4:** Differences in clinical outcomes between pre- and post-MPH treatment conditions.

	**T/T genotype group**		**G allele carrier group**		***P*** **-values**	
	**Pre-MPH Mean (SD)**	**Post-MPH_**1m**_ Mean (SD)**	**Pre-MPH Mean (SD)**	**Post-PH_**1m**_ Mean (SD)**	**T/T Pre vs. post**	**T/G Pre vs. post**
SNAP-IV IA	1.95 (0.42)	1.22 (0.52)	1.93 (0.33)	1.43 (0.81)	0.001^**^	0.06
SNAP-IV IH	1.50 (0.60)	1.03 (0.62)	1.14 (0.68)	1.03 (0.54)	0.003^**^	0.65
SNAP-IV ODD	1.50 (0.65)	0.97 (0.30)	1.02 (0.66)	0.85 (0.37)	**0.001** ^**^	0.54

*MPH, methylphenidate; SNAP-IV IA, inattention subscale scores; SNAP-IV IH, hyperactivity subscale scores; SNAP-IV ODD, oppositional defiance*.

** *p < 0.01*.

## Discussion

This study employed near-infrared spectroscopy imaging technology combined with the detection of *SNAP-25* gene *Mnl*I polymorphisms to explore the relationship between *SNAP-25* gene *Mnl*I polymorphisms and changes in brain function after MPH treatment in children with ADHD. Under the condition, where the baseline characteristics of the T/T genotype and G allele groups were consistent, we observed that SNAP-IV scores were significantly lower post-MPH treatment than pre-MPH treatment in the T/T genotype group. Moreover, in the T/T group, [Δavg oxy-Hb] in the DLPFC were also significantly increased after 4 weeks of MPH treatment. However, no significant differences were identified in SNAP-IV, go RT, go accuracy, or no-go accuracy between the pre- and post-MPH treatment conditions in the G allele group, indicating that longer MPH treatment may be more effective in the T/T genotype group than in the G allele group. Collectively, these findings indicate that *SNAP-25* gene *Mnl*I polymorphisms may be associated with hemodynamic changes in the DLPFC during longer MPH treatment in children with ADHD.

SNAP-25 is a key protein involved in the formation of soluble maleimide sensitive factor attachment protein receptor (SNARE) complexes in neurons. SNARE complexes play a crucial role in calcium-dependent endocytosis of synaptic vesicles, ensuring efficient neurotransmitter release and action potential propagation (Kádkov et al., 2019; Pozzi et al., 2019). These complexes are also necessary for learning, movement, memory formation, and normal brain function. Indeed, optimal levels of SNAP-25 are important for neurotransmission, and changes in SNAP-25 expression may contribute to the pathophysiology of various diseases, including Alzheimer's disease, schizophrenia, autism, and ADHD (Kim et al., 2007; Najera et al., 2019; Tang, 2021; Wang et al., 2021). Notably, genes for SNAP-25 and other SNARE complex proteins have been demonstrated to be associated with susceptibility and working memory in male patients with ADHD. Moreover, there is a significant difference in the distribution of associated SNP markers between patients with ADHD and controls (Gao et al., 2015), and *SNAP-25* gene *Mnl*I polymorphisms are associated with the severity of ADHD symptoms (Bidwell et al., 2017; González-Giraldo and Forero, 2020).

Herken et al. (2014) found that adult patients with the G/G genotype had higher Wender–Utah scores and higher scores in the 1st and 3rd components of the adult ADD/ADHD Scale, and Bidwell et al. (2017) showed a weak correlation between rs3746544 and ADHD in children. Notably, a meta-analysis of subgroups based on race showed that *Mnl*I polymorphisms were strongly associated with ADHD in Asian populations, but no significant association was identified in Caucasians (Ye et al., 2016), indicating that *Mnl*I polymorphisms are associated with ADHD depending on the genetic background of the population. Moreover, a US study of 165 preschoolers treated with MPH found that G allele carriers were 2–3 times more likely to present with irritability and sleep problems during MPH treatment than T allele carriers (McGough et al., 2006), while children with a homozygous T allele (T/T) genotype responded better to MPH treatment. Additionally, a Korean study of 139 children with ADHD reported that the rates of effective MPH treatment in the TT, TG, and GG genotype groups were 74.7, 72.5, and 33.3%, respectively (Song et al., 2014). In our study, SNAP-IV scores in the T/T genotype group were significantly lower after MPH treatment, and no significant differences were observed in SNAP-IV scores in the G allele genome between pre- and post-MPH treatment conditions. These findings suggest that *SNAP-25 Mnl*I polymorphisms in the Asian population might be associated with a response to longer MPH treatment, and the T/T genotype group might have better clinical outcomes, which might be related to changes in prefrontal hemodynamics.

Recent fMRI genetic studies have analyzed the effects of rs3746544 in Chinese Han children with ADHD. For example, resting-state fMRI studies showed that the regional homogeneity of the default mode network and working memory index were higher in the TT group than in the TG group (Fang et al., 2019), and G allele carriers had higher voxel-wise concordance in the right anterior central gyrus, superior frontal gyrus, posterior central gyrus, and middle frontal gyrus than those with TT homozygotes (Yang et al., 2020). In our study, fNIRS revealed that in the go/no-go task, [Δavg oxy-Hb] in the DLPFC in the T/T group were significantly increased post- MPH_4w_ treatment, whereas no significant changes in [Δavg oxy-Hb] were observed after treatment in the G allele group. Notably, the go/no-go task is known to activate the bilateral DLPFC (Liddle et al., 2001), and the activation may be modulated by the *SNAP-25 Mnl*I polymorphisms. Regional brain activation is accompanied by increases in regional cerebral blood flow and the regional cerebral metabolic rate of oxygen, and high oxygenated hemoglobin (HbO_2_) and low deoxygenated hemoglobin (HHb) levels may be associated with neurovascular coupling and increased blood flow to effectively carry hemoglobin from activated brain regions (Schroeter et al., 2002). However, the directions of the changes in oxy-Hb are always the same as that of the changes in cerebral blood flow, while the direction of the changes in deoxy-Hb is determined by changes in the venous blood oxygenation and volume (Hoshi, 2016). These findings suggest that oxy-Hb is the most sensitive indicator of changes in regional cerebral blood flow in NIRS measurements, and *SNAP-25* gene *Mnl*I polymorphisms affect regional cerebral blood flow and neurovascular coupling in the prefrontal cortex of children with ADHD, thus affecting their response to MPH treatment.

The go/no-go task is one of the most commonly used experimental paradigms to evaluate response inhibition (Aron and Poldrack, 2005). In this study, no significant differences were observed in go accuracy, no-go accuracy, or go RTs between the T/T genotype and G allele groups in the pre-MPH or post-MPH treatment condition. In the TT group, there were no significant changes in the go accuracy, no-go accuracy, and go RTs, but SNAP-IV scores were significantly decreased. However, usually, there are no correlations between the findings in the behavioral tasks and the questionnaires measuring impulsivity (Asahi et al., 2004; Claes et al., 2006). A possible explanation for these contradictory results might be that the behavioral tasks measure the inhibitory control of a specific facet of inhibition at a single point of time, while the scales rate general behaviors across different situations (Clark et al., 2009; Sánchez-Kuhn et al., 2017). In our study, fNIRS revealed the regional brain activation (DLPFC) in the go/no-go task in the T/T group and decreased SNAP-IV scores in the T/T group post- MPH_4w_ treatment. Therefore, fNIRS data during the go/no-go tasks combined with *SNAP-25 MnlI* polymorphism analysis in children with ADHD may be an effective biomarker to evaluate the effects of MPH treatment.MPH (Concerta) proved to have a predictable acute and chronic efficacy after a single administration (Swanson et al., 2002), in our study, the monitoring data of post-MPH_1.5h_ treatment reflects the acute effects of MPH, and the monitoring data of post-MPH_4w_ treatment reflects the superimposition of the chronic and acute effects of MPH. There were no significant changes in the brain function between pre-MPH and post-MPH_1.5h_ treatment or post-MPH_1.5h_ and post-MPH_4w_ treatment in both genotype groups, while there were significant changes in brain function between pre-MPH and post-MPH_4w_ treatment only in the T/T genotype group, indicating that acute or chronic effects of MPH alone hardly affect brain function, only the superimposition of the chronic and the acute effects of MPH can cause changes in brain function.

The results of our research were inconsistent with some of previous researches. For example, Monden et al. (2012a,b) found that after 1.5 h of MPH intake, significant MPH-elicited activation (oxygenated hemoglobin signal increase) was detected in the right lateral prefrontal cortex, but all children enrolled in the study had received prior MPH therapy for 1 week to 3.6 years. Although these children underwent a 24-h washout period at the time of the study, It has been suggested that that previous treatment of MPH may have affected brain function (Ishii-Takahashi et al., 2015). In addition, the dosage of MPH may also affect the changes of brain function A recent meta-analysis on the effects of MPH on various neuropsychological tasks found that higher doses of MPH resulted in greater improvements for some tasks than lower doses (Pietrzak et al., 2006). Moreover, With or without comorbidities (Ishii-Takahashi et al., 2014; Bruder et al., 2017) and different cognitive tasks may also influence the outcome of changes in brain function (Comalli et al., 1962; Nakanishi et al., 2017).

This study has several limitations. First, this was a non-randomized controlled study; hence, we could not assess potential placebo effects. Second, this is a pilot study, the sample size was small. In particular, the number of G allele carriers was only 11, which might have reduced test efficiency. Further large-sample randomized controlled studies are warranted to validate our findings. Third, the age range of participants (years) was relatively wide. The maturation of the prefrontal cortex has a protracted, step-wise pattern, which can lead to a wide variability of prefrontal activation patterns across ages (Yaple and Arsalidou, 2018). Although most previous ADHD studies were conducted with similar age ranges to ours, it would be better to narrow the age range to obtain more accurate results on prefrontal function in children. Fourth, our study only tested inhibitory function using the go/no-go paradigm but did not evaluate other working memories, such as conversion memory. In addition, limited brain regions were analyzed in this study; thus, our findings could not fully reflect changes in the function of the entire frontal lobe; further studies analyzing more prefrontal regions are needed.

In conclusion, our study demonstrates that *SNAP-25 Mnl*I polymorphisms may be associated with the response to MPH treatment and may affect neurovascular coupling in the prefrontal cortex in children with ADHD, thereby affecting their response to MPH treatment. Moreover, Our preliminary study indicate that near-infrared brain function monitoring data during the go/no-go tasks combined with *SNAP-25 Mnl*I polymorphism analysis may be a useful biomarker to evaluate the effects of MPH treatment in children with ADHD.

## Data Availability Statement

The raw data supporting the conclusions of this article will be made available by the authors, without undue reservation.

## Ethics Statement

The studies involving human participants were reviewed and approved by the Ethics Committee of Shanghai Children's Medical Center, School of Medicine, Shanghai Jiao Tong University (SCMCIRB-K2021045-1). Written informed consent to participate in this study was provided by the participants' legal guardian/next of kin.

## Author Contributions

JL devised the project under the supervision of Y-WZ. JL and W-JY enrolled the patients. JL, W-JY, and X-XT performed neuropsychological examinations. JL, W-JY, and YW performed data collection and analysis. JL wrote the first draft. W-JY, YW, and Y-WZ revised the manuscript. All the authors approved the final version of the manuscript.

## Funding

This study was supported by Shanghai Science and Technology Commission of Shanghai Municipality (21Y11907400, 17411965300), Shanghai Municipal Science and Technology Commission, early diagnosis and intervention of children with ASD based on brain function and VR technology (21Y11905500), and multi-disciplinary project cultivation fund of Shanghai Jiao Tong University (YG2021QN111).

## Conflict of Interest

The authors declare that the research was conducted in the absence of any commercial or financial relationships that could be construed as a potential conflict of interest.

## Publisher's Note

All claims expressed in this article are solely those of the authors and do not necessarily represent those of their affiliated organizations, or those of the publisher, the editors and the reviewers. Any product that may be evaluated in this article, or claim that may be made by its manufacturer, is not guaranteed or endorsed by the publisher.
